# Supplementary material for: Impact of adherence and stringency on the effectiveness of lockdown measures: A modelling study
Source: PLoS One. 2025 Dec 19;20(12):e0338818. doi: 10.1371/journal.pone.0338818 (PMC12716724; doi:10.1371/journal.pone.0338818)
Supplement: S1 Appendix — (PDF) [file pone.0338818.s001.pdf]

Supporting information file 1 — Impact of adherence and stringency on the effectiveness of lockdown measures: a modelling study

Joren Brunekreef [1◑¤], Alexandra Teslya [1◑], Vincent Buskens [2,3], Hendrik Nunner [3, 4], Mirjam Kretzschmar [1, 3, 5*]

1 Julius Center for Health Sciences and Primary Care, University Medical Center Utrecht, Utrecht University, Utrecht, The Netherlands
2 Department of Sociology / ICS, Utrecht University, Utrecht, The Netherlands
3 Center for Complex Systems Studies, Utrecht University, Utrecht, The Netherlands
4 Institute for Multimedia and Interactive Systems, University of Lübeck, Germany
5 Interdisciplinary Center for the Mathematical Modeling of Infectious Disease Dynamics (IMMIDD), University of Münster, Germany

◑These authors contributed equally to this work.
¤Current Address: Netherlands Cancer Institute, Amsterdam, The Netherlands
* m.e.e.kretzschmar@umcutrecht.nl

S1 Appendix: Technical Details of Simulation Model

Overview

The model captures the dynamics of infection spread and health opinion competition in a fixed-size population. Infection transmission dynamics is described by Susceptible-Infected-Removed (SIR) framework whereupon each individual belongs to one of three classes. Susceptible individuals who come into contact with infectious individuals can become infected. If transmission of infection has taken place, individuals become infectious. After a period of time infectious individuals recover and become immune to further acquisition of infection. There are two mutually exclusive health opinions circulating in the population: health-positive, $\bigoplus$, and health-neutral, $\bigotimes$. Each individual holds one of two opinions. Individuals can switch opinion upon interacting with their peers who hold the opposite opinion. The more such individuals the faster this switch will occur. Switching of opinions can occur in a disease-free population.

Infection transmission dynamics and opinion switching are coupled via two mechanisms. Presence of the infection as captured by the global prevalence leads to increase in the switch rate to health-positive opinion. If in a response to the outbreak a lockdown is initiated, switch rate to health-neutral opinion increases, such that the increase is positively correlated to stringency and the duration of the lockdown.

Below we describe mathematical formulations and methods used to simulate the described dynamics.

Table A. Model parameters

Symbol	Description	Value(s), unit
β, K	Watts-Strogatz algorithm [1] rewiring probability and mean degree, respectively	$\beta = 0.08$<br>$K = 14$
γ	Disease recovery rate	1.0, week^{-1}
$c_{\text{phys}}(j, j')$	Physical contact rate between individuals j, j'	7.0, week^{-1}
$c_{\text{inf}}(j, j')$	Information contact rate between individuals j, j'	10.0, , week^{-1}
p	Opinion switch probability per information contact event	0.04
k, θ	Opinion switch propensity function shape parameters	$k = 1.8$<br>$\theta = 7.0$
$\epsilon_{\oplus}, \epsilon_{\otimes}$	Infection probability for opinion $\bigoplus$, $\bigotimes$ individuals per physical contact event with infected peer (of arbitrary opinion)	$\epsilon_{\oplus} = 2.0 \times 10^{-2}$<br>$\epsilon_{\otimes} = 3.5 \times 10^{-2}$
C_{fat}	Weight factor of lockdown fatigue contribution to opinion switch propensity	5.0×10^{-2}
C_{hs}	Weight factor of prevalence related contribution to opinion switch propensity	2.0
f_s	Threshold prevalence for lockdown initiation	$(0.5, 1.0, 2.0, 3.5, 5.0) \times 10^{-2}$
f_e	Threshold prevalence for lockdown lifting	1.5×10^{-3}
q	Lockdown stringency	0.00, 0.25, 0.5, 0.75, 1.00
α	Lockdown adherence for opinion $\bigotimes$ individuals (high, medium, low)	1.0, 0.5, 0.0

Networks

In the context of the model definition, individuals are represented as nodes in a multiplex network. The first layer of the network (physical network) corresponds to

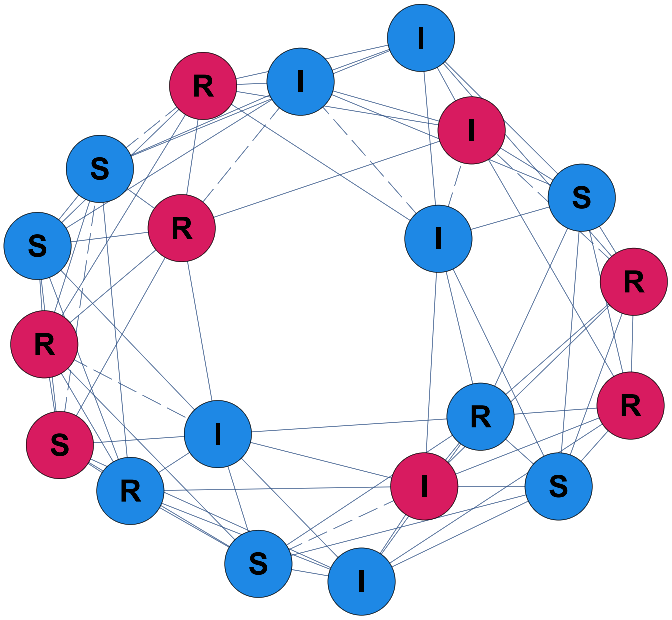

Fig S1. A small-world network consisting of 20 nodes. In the example shown, the mean degree is $K = 4$ and the rewiring probability for the Watts-Strogatz algorithm is $\beta = 0.1$. Letters indicate disease status (Susceptible, Infected, or Recovered), and colors represent opinion state (blue for health-positive, red for health-neutral). Solid lines allow for both informational and physical contact, whereas dashed lines allow for informational contact only, preventing disease transmission through such an edge.

physical interactions which can result in infection acquisition. The second layer (social network) represent social interactions necessary for opinion competition dynamics.

In each network, connection of two nodes(j and j') by an edge represents an interaction that persists in time with a certain frequency. In the physical network the frequency of interactions (physical contact rate) is denoted by $c_{\text{phys}}(j, j')$, in social network (information contact rate) it is denoted by $c_{\text{inf}}(j, j')$.

At the start of the simulation, both networks are identical. However, as an outbreak develops and lockdowns are initiated, the physical network changes its structure. Once a lockdown is relaxed, the physical network reverts to its pre-lockdown state. The networks are small-world networks created using the Watts-Strogatz algorithm [1], which starts with a ring lattice with average degree $K = 14$ and rewires network edges with probability $\beta = 0.08$ per edge. The resulting networks have clustering coefficient 0.54 and average path length 5.4.

We present a schematic representation of a small-world population network in Fig. S1. The label of a node indicates the disease state of an individual as either Susceptible (S), Infected (I), or Recovered (R). The color of the node indicates the opinion status, being either health-positive (blue) or health-neutral (red). Solid lines mean that the contact edge between two individuals is of both a *physical* and *informational* nature, whereas a dashed line indicates that the physical nature of the edge is switched off temporarily due to an active lockdown state. Disease transmission can only occur through solid lines.

Simulation algorithm

The dynamics of infection transmission and opinion switching processes are modeled using the Gillespie Algorithm [2, 3] as follows.

The state of the population, denoted X, which encompasses both epidemiological state distribution and opinion distribution, can be modified by an event l. This event might either cause a change in individual's epidemiological status or a switch of their opinion. Each event has an associated propensity function $\phi_l(X)$ defined as follows. At a time t, given the state of the system $X(t)$, an event l has probability of occurring in the interval $[t+\tau, t+\tau+\Delta\tau)$ given by

$$p(\tau, l|X(t)) = \phi_l(X(t)) \exp(-\phi_0\,(X(t))\,\tau)\Delta\tau \tag{S1}$$

where

$$\phi_0(X(t)) = \sum_l \phi_l(X(t)) \tag{S2}$$

where l iterates over the set of all possible events.

Thus, at each time point, given the current state of the system, we calculate the propensities for each possible event to take place. Then, using the joint probability distribution given by Eq. (S1) and Eq. (S2), an event that occurs and the time of its occurrence are determined. The system is updated accordingly and the step is completed. Note that given a model population size N, in each step we need to calculate propensities for $2N$ events.

Epidemiological state switch propensities

In the context of infection transmission dynamics, each individual can experience one of two possible events: infection ($S \to I$) or recovery ($I \to R$) with transition rates ϕ_{SI} and ϕ_{IR}, respectively. Note that at any given time, an individual can undergo either one or the other event.

The propensity of individual j holding opinion $\mathrm{Op}(j) \in \{\bigoplus, \bigotimes\}$ with the set of infected peers on the physical interaction network given by $\mathrm{IPN}(j)$ to experience event $S \to I$ is defined by

$$\phi_{SI}(j) = \epsilon_{\mathrm{Op}(j)} \sum_{j' \in \mathrm{IPN}(j)} c_{\mathrm{phys}}(j, j'), \tag{S3}$$

where $\epsilon_{\mathrm{Op}(j)}$ is the probability for infection acquisition per contact with an infected peer. The infection acquisition probability $\epsilon_{\mathrm{Op}(j)}$ is lower for individuals holding health-positive opinion $\bigoplus$ than for individuals with health-neutral opinion $\bigotimes$, and these probabilities remain fixed throughout the simulation.

Contact rates c_{phys} are a subject to presence of the lockdown. During a lockdown, a proportion $q \in [0, 1]$ of contact rates will be set to zero. This implements the effect of lockdowns on the contact rates and subsequently on transmission of infection. When the lockdown ends, the contact rates revert to their original value.

In the model, health-neutral individuals can partially ignore lockdown rules, so the probability that contact rates between two such individuals reduce to zero is smaller than for contact rates between people where at least one of the two peers is of health-positive opinion. We define the parameter $\alpha \in [0, 1]$ to encode the resistance of health-neutral individuals to lockdown rules, such that the lower is the value of α the less likely individuals to comply with lockdown.

Therefore, to calculate contact rates (and thus, the propensities of infection transmission) in the conditions of lockdown, we iterate through all edges jj' of the

physical interaction network, and set the contact rate (weight) of the edge to zero with probability $p_{jj'}$, which we define as

$$p_{jj'} = \begin{cases} (1-\alpha)q, & \text{if } \mathrm{Op}(j) = \mathrm{Op}(j') = B \\ q, & \text{otherwise} \end{cases} \tag{S4}$$

The $I \to R$ transition only depends on the recovery rate γ, and is simply written

$$\phi_{IR}(j) = \gamma. \tag{S5}$$

Opinion state switch propensities

Opinion switches can take place from $\bigoplus$ to $\bigotimes$ and from $\bigotimes$ to $\bigoplus$. The model incorporates opinion switching in the disease-free regime based on information exchange with peers of opposite opinion, as described in [4]. For an individual i, denote the fraction of their peers with health opinion $l \in \{\bigoplus, \bigotimes\}$ by $n_l(j)$. Then the rates with which individuals switch their opinion are given by the following functions

$$\tilde{\phi}_{\bigoplus\bigotimes}(j) = c_{\inf} \frac{p_{\bigotimes} n_{\bigotimes}(j)^k}{1 + \theta_{\bigotimes} n_{\bigotimes}(j)^k}, \quad \text{from } \bigoplus \text{ to } \bigotimes \tag{S6}$$

$$\tilde{\phi}_{\bigotimes\bigoplus}(j) = c_{\inf} \frac{p_{\bigoplus} n_{\bigoplus}(j)^k}{1 + \theta_{\bigoplus} n_{\bigoplus}(j)^k}, \quad \text{from } \bigotimes \text{ to } \bigoplus \tag{S7}$$

where p_l is the probability of switching opinion per contact and θ_l and k are parameters that determine the shape of the switch rate function. By adjusting these shape parameters, we can continuously transform the switch rate propensity from a linear dependence on n_l to a function that saturates at a given threshold, and finally to a sigmoid shape. We present examples of several possible choices of the shape parameters in Fig. S2. In the simulations, we have set k and θ such that switch rate function have sigmoidal shape which results in a possibility of stable co-existence of two opinions in the population. Probabilities of switching opinion per contact, p, were set to the same value for both opinions, and the initial proportion of individuals holding either opinion was set to 0.5. These settings, in the absence of infection, lead to a quasi-steady distribution of opinions of around $(0.5, 0.5)$. Note that for simplicity we have implicitly assumed information contact rates of all edges to take the same value $c_{\inf}$.

To model feedback relationship between infection spread, measures aiming to control it, and opinion competition, we modified the opinion switch propensity functions by including additional terms. To reflect the 'lockdown-fatigue' effect, which mimics people gradually getting weary of being in a lockdown regime and therefore, becoming more inclined to switch to a health-neutral opinion, we introduced a second term in the $\bigoplus \to \bigotimes$ opinion switch propensity function. We assume that the fatigue effect is stronger for more stringent and longer lockdowns. To capture this effect in the model we define a quantity $\xi_{\bigoplus\bigotimes}(t_l, q)$, where t_l is the duration of the lockdown at time t (i.e., the time since initiation of the lockdown measures until the current point t) and q is the lockdown stringency. We assume that ξ is an increasing function of both t_l and q. In the simulations presented here, we defined ξ as

$$\xi_{\bigoplus\bigotimes}(t_l, q) = C_{lf}\, t_l\, q. \tag{S8}$$

The parameter C_{lf} determines the relative weight of the lockdown-fatigue function with respect to the opinion switch based on local opinion distributions, $\tilde{\phi}$.

Combining the two processes yields the propensity $\phi_{\bigoplus\bigotimes}$ for an individual j with opinion $\bigoplus$ to change their opinion to $\bigotimes$ as follows:

$$\phi_{\bigoplus\bigotimes}(j) = \tilde{\phi}_{\bigoplus\bigotimes}(j) + \xi_{\bigoplus\bigotimes}(t_l, q). \tag{S9}$$

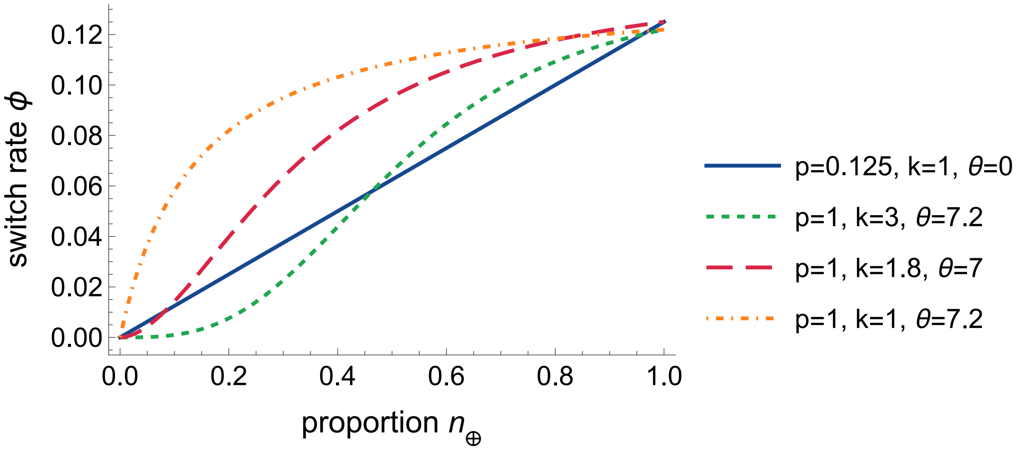

Fig S2. Examples of opinion switch propensity functions. By varying the shape parameters, we can model linear (blue, solid), saturating (orange, dash-dotted), and sigmoidal (red/green dashed) dependence of switch propensities on the independent variable. The green dashed sigmoid has a delayed growth onset as compared to the red dashed sigmoid. In our simulations, we have used function whose shape is similar to the red dashed curve.

Similarly, to model the 'health-scare' dynamic, we introduce an additional term in the $\otimes\rightarrow\oplus$ opinion switch rate. 'Health-scare' refers to the phenomenon when people are influenced to adopt a health-positive opinion when the population-level disease prevalence increases. Therefore, during an outbreak the switch propensity $\otimes\rightarrow\oplus$ depends on the global disease prevalence P, with the functional dependence of the propensity for a health-scare-induced opinion given by

$$\xi_{\otimes\oplus}(P) = C_{hs}P. \tag{S10}$$

The complete expression for propensity $\phi_{\otimes\oplus}$ for an opinion switch from $\otimes$ to $\oplus$ is then

$$\phi_{\otimes\oplus}(j) = \tilde{\phi}_{\otimes\oplus}(j) + \xi_{\otimes\oplus}(P). \tag{S11}$$

References

1. Watts D, Strogatz S. Collective dynamics of 'small-world'networks. Nature. 1998;393(6684):440-2. doi:10.1038/30918.

2. Gillespie D. A general method for numerically simulating the stochastic time evolution of coupled chemical reactions. Journal of computational physics. 1976;22(4):403-34. doi:10.1016/0021-9991(76)90041-3.

3. Gillespie DT. Stochastic simulation of chemical kinetics. Annu Rev Phys Chem. 2007;58:35-55. doi:10.1146/annurev.physchem.58.032806.104637.

4. Teslya A, Nunner H, Buskens V, Kretzschmar ME. The effect of competition between health opinions on epidemic dynamics. PNAS Nexus. 2022 Nov;1(5):pgac260. doi:10.1093/pnasnexus/pgac260.
