# Supplementary material for: Impact of adherence and stringency on the effectiveness of lockdown measures: A modelling study
Source: PLoS One. 2025 Dec 19;20(12):e0338818. doi: 10.1371/journal.pone.0338818 (PMC12716724; doi:10.1371/journal.pone.0338818)
Supplement: S2 Appendix — (PDF) [file pone.0338818.s002.pdf]

Supporting information file 2 — Impact of adherence and stringency on the effectiveness of lockdown measures: a modelling study

Joren Brunekreef [1◑¤], Alexandra Teslya [1◑], Vincent Buskens [2,3], Hendrik Nunner [3, 4], Mirjam Kretzschmar [1, 3, 5*]

1 Julius Center for Health Sciences and Primary Care, University Medical Center Utrecht, Utrecht University, Utrecht, The Netherlands
2 Department of Sociology / ICS, Utrecht University, Utrecht, The Netherlands
3 Center for Complex Systems Studies, Utrecht University, Utrecht, The Netherlands
4 Institute for Multimedia and Interactive Systems, University of Lübeck, Germany
5 Interdisciplinary Center for the Mathematical Modeling of Infectious Disease Dynamics (IMMIDD), University of Münster, Germany

◑These authors contributed equally to this work.
¤Current Address: Netherlands Cancer Institute, Amsterdam, The Netherlands
* m.e.e.kretzschmar@umcutrecht.nl

S2 Appendix: Parameter Calibration

The values of the infection probabilities $\epsilon_{\oplus}, \epsilon_{\otimes}$ and the opinion switch weight factors $C_{\text{fat}}, C_{\text{hs}}$ were fixed sequentially using a calibration procedure. For each parameter, we outlined heuristic conditions that we wanted our model to satisfy. We then performed 500 simulation runs for a range of candidate values of that parameter, and picked the value for which the appropriate heuristic condition was best met.

The first parameter to be calibrated was the probability of infection for health-positive individuals $\epsilon_{\oplus}$. The rationale for choosing the value of this parameter was that the infectious disease introduced to a population consisting solely of health-positive individuals should, in most cases, stay slightly below the epidemic threshold, meaning that the effective reproduction number is below 1. This means that the epidemic does not take off, but goes extinct after a short time. In the calibration runs for this parameter, no lockdowns were imposed, so that all individuals remain in the $\bigoplus$ state (cf. S1 Appendix Eq. S7). We used the values $\epsilon_{\oplus} \in \{0.015, 0.02, 0.025, 0.03, 0.035\}$ as the candidate range. As seen in Fig. S3, the choice $\epsilon_{\oplus} = 0.02$ leads to a scenario where the median prevalence is projected to be small with the upper 95% quantile indicating that even in the case of the largest expected outbreak the prevalence will not reach high values. The median epidemic size is roughly the same for $\epsilon_{\oplus} = 0.025$, but the 95% quantiles now show larger outbreaks. Therefore, we fixed the infection probability for health-positive individuals to $\epsilon_{\oplus} = 0.02$.

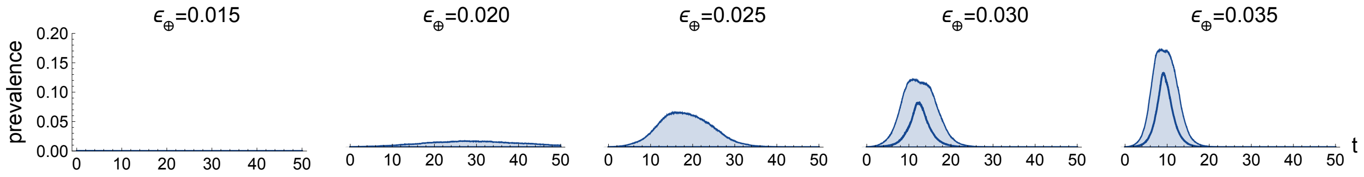

Fig S3. Median and 95% CI of the prevalence over time (t, in weeks) for different values of the infection probability $\epsilon_{\oplus}$ for health-positive individuals.

Next, we calibrated the probability of infection probability for health-neutral individuals $\epsilon_{\otimes}$, setting $\epsilon_{\oplus}$ to its fixed value 0.02 and the health-scare opinion switch weight factor C_{hs} to zero. We assumed that in a population with equal numbers of health-neutral and health-positive individuals larger outbreaks of the infectious disease should be possible. We tested the range $\epsilon_{\otimes} \in \{0.025, 0.03, 0.035\}$ and chose $\epsilon_{\otimes} = 0.035$ (see Fig. S4). Note that opinion switches do occur in these simulation runs, but since $C_{\text{hs}} = 0$ and no lockdowns are imposed, the opinion switches are only induced by the local opinion distribution and the population-level opinion distribution remains approximately constant.

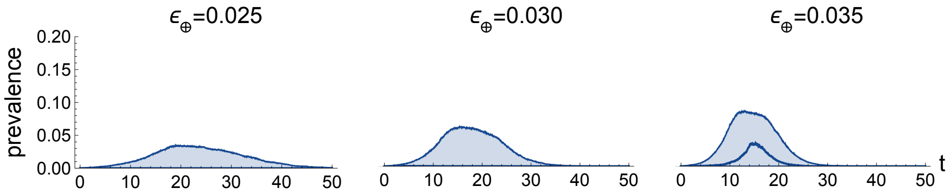

Fig S4. Median and 95% CI of the prevalence over time (t, in weeks) for different values of the infection probability $\epsilon_{\otimes}$ for health-neutral individuals.

The next parameter to be calibrated was the weight factor C_{hs} for the health-scare function $\xi_{\bigotimes\bigoplus}$ in the opinion switch propensity from health-neutral to health-positive

(cf. S1 Appendix equations S10 and S11). The rationale here was that the population should undergo a sizeable shift towards a health-positive opinion during an outbreak, but this should not lead to extinction of the health-neutral opinion. As shown in Fig. S5, the value $C_{\mathrm{hs}} = 2.0$ is a choice fulfilling this requirement. The rightmost graph shows that the health-neutral opinion goes extinct in the median scenario for the choice $C_{\mathrm{hs}} = 8.0$.

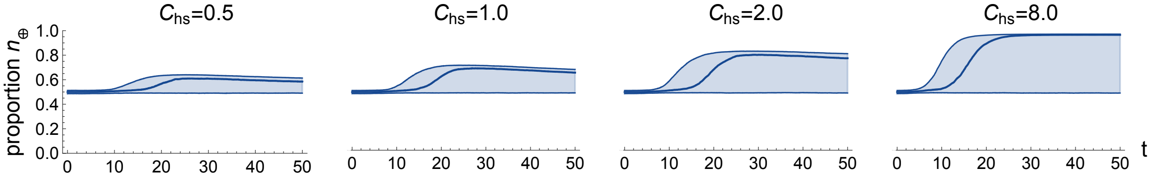

Fig S5. Median and 95% CI of the proportion of health-positive individuals over time (t, in weeks) for different values of the health-scare weight factor C_{hs}.

Finally, we calibrated the weight factor C_{fat} for the lockdown fatigue function, which increases the propensity for health-positive individuals to switch to a health-neutral opinion (cf. S1 Appendix equations S8 and S9). Using the previously described method to calibrate C_{hs}, we chose a value of C_{fat} for which the population makes a strong shift towards a health-neutral opinion. The graphs shown in Fig. S6 support our choice of $C_{\mathrm{fat}} = 0.05$ for this parameter.

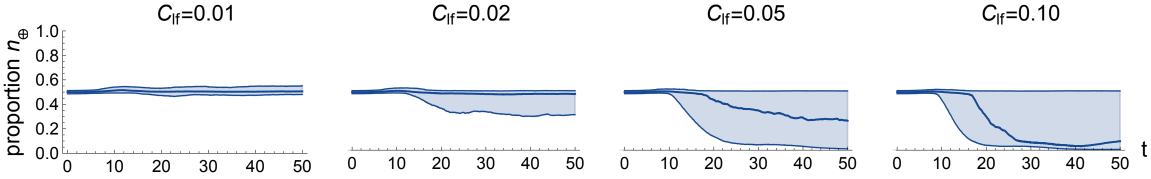

Fig S6. Median and 95% CI of the proportion of health-positive individuals over time (t, in weeks) for different values of the lockdown fatigue weight factor C_{fat}.
