# Supplementary material for: Impact of adherence and stringency on the effectiveness of lockdown measures: A modelling study
Source: PLoS One. 2025 Dec 19;20(12):e0338818. doi: 10.1371/journal.pone.0338818 (PMC12716724; doi:10.1371/journal.pone.0338818)
Supplement: S3 Appendix — (PDF) [file pone.0338818.s003.pdf]

Supporting information file 3 — Impact of adherence and stringency on the effectiveness of lockdown measures: a modelling study

Joren Brunekreef [1◑¤], Alexandra Teslya [1◑], Vincent Buskens [2,3], Hendrik Nunner [3, 4], Mirjam Kretzschmar [1, 3, 5*]

1 Julius Center for Health Sciences and Primary Care, University Medical Center Utrecht, Utrecht University, Utrecht, The Netherlands
2 Department of Sociology / ICS, Utrecht University, Utrecht, The Netherlands
3 Center for Complex Systems Studies, Utrecht University, Utrecht, The Netherlands
4 Institute for Multimedia and Interactive Systems, University of Lübeck, Germany
5 Interdisciplinary Center for the Mathematical Modeling of Infectious Disease Dynamics (IMMIDD), University of Münster, Germany

◑These authors contributed equally to this work.
¤Current Address: Netherlands Cancer Institute, Amsterdam, The Netherlands
* m.e.e.kretzschmar@umcutrecht.nl

S3 Appendix: Additional Results

In Fig. S7 we show the final proportion of health-neutral individuals (FPHN) for each individual simulation run, plotted against the total number of weeks during which there was an active lockdown in that run. This provides further insight into the mutual interaction between higher proportions of health-neutral individuals and higher disease prevalences. In this figure, the detrimential effect on the health opinion landscape of a non-effective lockdown is evident, regardless of whether it was due to low stringency or to low adherence. In this scenario, the lockdowns result in a significant increase in FPHN, not only compared to the baseline scenarios, but also compared to scenarios where lockdowns were more effective and subsequently shorter.

The results of additional sensitivity analyses for the stringency parameter q and the adherence parameter α can be found on this project's GitHub page [1].

References

1. Brunekreef J, Teslya A. JorenB/infections-lockdowns-opinions; 2024. Available from: `https://github.com/JorenB/infections-lockdowns-opinions`.

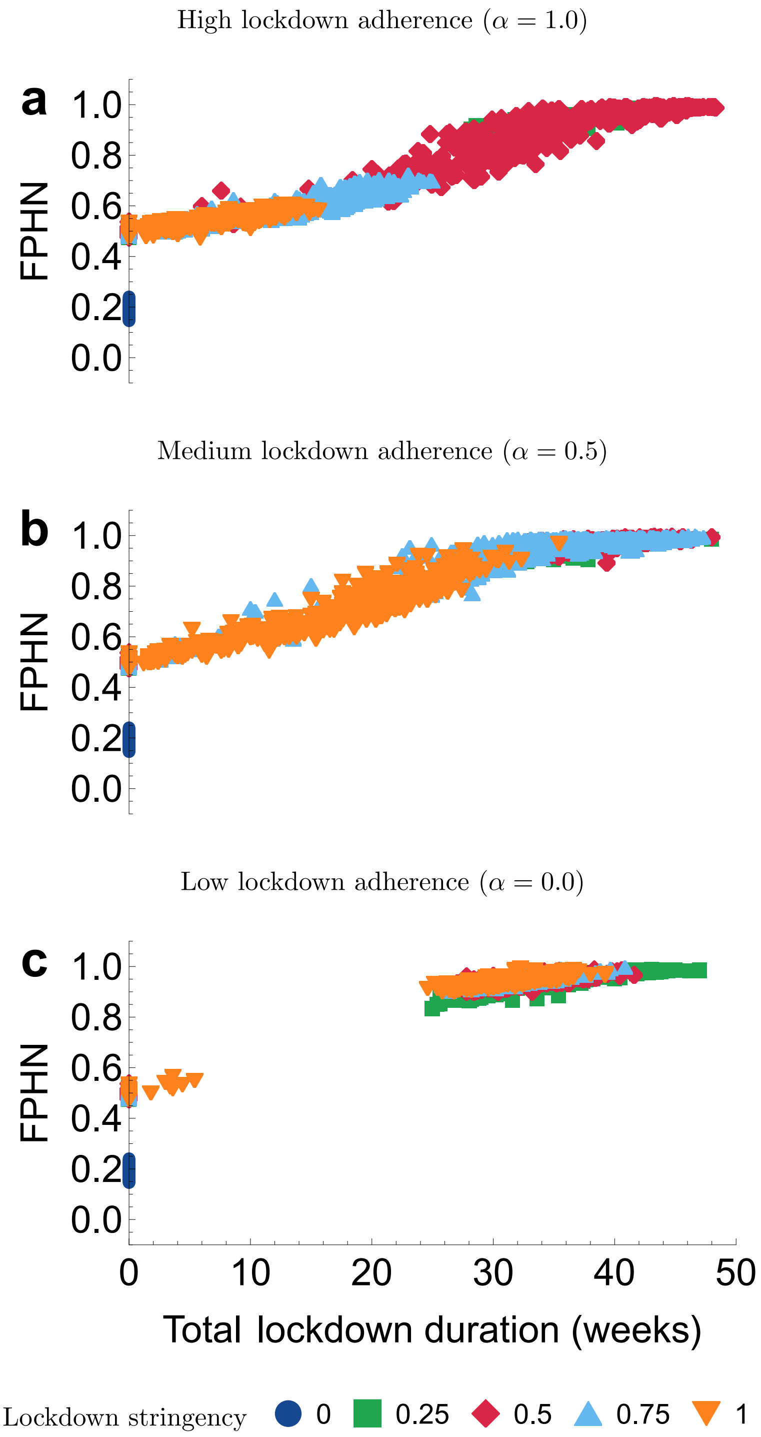

Fig S7. Opinion distribution and lockdown duration. The relative final proportion of health-neutral individuals (FPHN) versus the total lockdown duration, across various levels of lockdown adherence parameter $\alpha = 1.0, 0.5, 0.0$. The FPHN is reported relative to the mean FPHN in the scenario where no lockdown is implemented ($q = 0$). Each marker corresponds to a single simulation run. The threshold prevalence for lockdown initiation is fixed at $f_s = 0.005$.
